# Prevalence of Neck Pain and Associated Factors with Personal Characteristics, Physical Workloads and Psychosocial among Male Rubber Workers in FELDA Settlement Malaysia

**DOI:** 10.5539/gjhs.v4n1p94

**Published:** 2012-01-01

**Authors:** Chow Li Shan, Mohd Yusoff Bin Adon, Anita Binti Abd Rahman, Syed Tajuddin Syed Hassan, Kamal Bin Ismail

**Affiliations:** Department of Community Health Faculty of Medicine and Health Sciences Universiti Putra Malaysia Tel: 012-546-5938 E-mail: cherrylishan@yahoo.com; Associate Prof. Institute Medical Center Jalan Pahang, 50588 Kuala Lumpur Tel: 60-326-162-666 E-mail: yusoff@imr.gov.my; Department of Community Health Faculty of Medicine and Health Sciences Universiti Putra Malaysia Tel: 60-389-472-409 E-mail: anitaar@medic.upm.edu.my; Department of Medicine Faculty of Medicine and Health Sciences Universiti Putra Malaysia Tel: 60-389-472-421 E-mail: tajuddin@medic.upm.edu.my; Department of Community Health Faculty of Medicine and Health Sciences Universiti Putra Malaysia Tel: 60-389-472-394 E-mail: kamal838@gmail.com

**Keywords:** Neck pain, Musculoskeletal symptoms, Physical workloads, Job insecurity, Rubber workers

## Abstract

Rubber tapping processes posed potential risk of various health problems among rubber workers. It ranges from simple musculoskeletal aches to more serious and complicated structural damage to bone, muscles, tendons and nerves of musculoskeletal system. These health problems might be linked directly to the arduous demands of farm labor.

**Objectives:**

A cross-sectional study was conducted to determine the prevalence of neck pain (NP) and musculoskeletal symptoms (MSS) and its association with personal characteristics, physical workloads and psychosocial factors among rubber workers.

**Methods:**

Stratified random sampling method was adopted and a total of 419 rubber workers in FELDA’s scheme Malaysia participated in this study. Data was collected through face to face interview using modified Standardized Nordic Questionnaire (SNQ) and Job Content Questionnaire (JCQ).

**Results:**

The results revealed the prevalence of NP was 59.9% and weak correlation with age (ρ= -0.184, *p*= 0.001) and a positive weak correlation with working hours per day (ρ= 0.099, *p*= 0.043) significantly. All physical workloads (neck flexion or rotation, awkward postures, repetitive motion and static postures) had significant weak to moderate positive correlation with NP (*p*<0.05). Job insecurity was found to have weak and positive correlation with NP (*p*<0.05). Binary logistic regression analysis showed risk factors for NP were decreased with age (OR= 3.92, 95% CI 1.61 – 9.58, p=0.003), increase in neck flexion or rotation (OR= 9.52, 95% CI 5.55 – 16.32, p= 0.001), awkward postures (OR=2.23, 95% CI 1.29 – 3.86, p= 0.004) and static postures (OR= 1.86, 95% CI 1.10 – 3.14, p= 0.021).

**Conclusion:**

This study showed that high prevalence of NP was associated with neck flexion or rotation, awkward and static postures.

## 1. Introduction

In Malaysia, agriculture is being promoted as the third engine of growth and modernization for poverty reduction as stated in its latest ninth- 5-year development plan (Ninth Malaysian Plan, 2006). Rubber industry is the second largest agriculture industries in Malaysia with the land uses of 1250000 hectare which was 19.58% of the overall land uses for agriculture (MOA and MPIC, 2005). However, farming and agriculture tasks are highly physical demanding, arduous and require extremely large of energy in performing their tasks. Hence, this poses farm workers at potential risk of health problems caused by physical hazards, chemical hazards, biological hazards, psychosocial hazards and ergonomic hazards. Ergonomic hazards poses variety of musculoskeletal symptoms (MSS) encompasses the neck, low back pain, osteoarthritis of the hip and knee, upper limb complaints and hand-arm vibration syndrome (MTUC, 1989; Walker-bone *et al.*, 2002).

Neck pain is among the commonest MSS faced by general population and more so among the rubber workers. FELDA (Federal Land Development Authority) is the world’s largest plantation operator with 811,140 hectares agriculture area. 722,946 hectares or 84.7 % of agricultural area are planted with oil palm, 84,496 hectares or 9.9 % are planted with rubber, 879 hectares or 0.1 % are planted with sugar cane and 2,819 hectares or 0.3 % are plantation such as timber trees, fruits and also used for research (FELDA Annual Report, 2008). FELDA has implemented replanting program in 42,910 hectares of its rubber plantation because most of rubber trees in FELDA are old as height of tapping trees increase, the tapping tasks prone to focus on upper limb mainly on neck region (FELDA Annual Report, 2008). A preliminary study of prevalence of MSS among Malaysian rubber tappers found that the prevalence of MSS within the last 12 months showed the highest for low back (74.4%) follow by shoulder (53.3%), neck (48.8%) and wrist/hand (48.8%), upper back (41.9%), elbow (39.5%), knee (39.5%) and ankle/foot (9.3%) (Asyraf *et al.*, 2007). In a study by Rosnah *et al*.(2007) on different occupation work posture risk analysis among oil palm plantation mechanical loader operators found that the prevalence of NP was highest with 84 respondents reported NP followed by knee pain (35 respondents), upper back pain (27 respondents) and ankle/feet pain, 24 respondents (Rosnah *et al.*, 2007).

Overall tasks of rubber tapping would expose rubber tappers to ergonomics risk factors such as repetitiveness, awkward postures, static muscle loading and forceful exertion (Asyraf *et al.*, 2007). Among the ergonomic factors present in rubber tapping process include age of the trees, height of tapping areas, number of area being tapped, uneven ground and technique of performing the tapping. However, the main risk factors for NP in rubber field are neck extension, twisted head, flexion of neck, awkward postures and repetitive moving of the head. In addition, psychosocial hazards such as low job dissatisfaction, supervisor rating, psychological demands, decision latitudes and social support were the factors to cause sick leaves or disability due to Musculoskeletal Disorders (MSDs) (Hartman *et al.*, 2006). These hazards were overlooked as the main cause of MSDs and neck pain among rubber tappers.

Ergonomic risk factors cause wide range of occupational related diseases among agriculture workers especially rubber plantation population and need extensive exploration in Malaysia. In view of limited study on prevalence of NP and MSDs among rubber workers in Malaysia, this study was attempted to determine the prevalence of NP and its association with personal characteristics, physical workloads and psychosocial factors.

## 2. Material and Methods

### 2.1 Study Design and Study Population

A cross-sectional study was conducted among rubber workers from October 2008 until May 2009 in six FELDA in Sembilan State, Malaysia using stratified random sampling in which sample was stratified and drawn from the overall total population in six’s FELDA with proportional. The inclusion criteria in this study was Malaysian rubber workers age from 39 to 66 years old without medical diseases or trauma affecting musculoskeletal system and accident in any part of the body as majority of the active rubber workers in FELDA are in this age of range in Malaysia.

Minimum calculated sample size based on Daniel (1999) with 49% (Asyraf *et al.*, 2007) prevalence of NP was 384 respondents with α = 5%, and margin error at 0.05. An additional of 10% of calculated sample size brought the total respondent to 423 workers. However, in this study 419 respondents out of 554 male rubber workers were eligible for further analysis.

### 2.2 Data Collection Material

A modified Standardized Nordic Questionnaire (SNQ) consists of four parts (i) socio-demographic characteristics, (ii) general questionnaire, (iii) detailed body part-specific symptoms and (iv) physical workload rating was used to determine prevalence of NP.

Socio-demographic part contain question on age, education level, height, weight, working hours per day, length of working, smoking habit and trees tapped per day. General questionnaire showed a body map of nine-anatomical body regions and asking about ache, pain, and discomfort for the last 12 months in each of the body regions and past 7 days discomfort. Rubber workers who faced 3 months continuous ache, pain and discomfort in last 12 months was included in the analysis instead of 7 days discomfort as 7 days may be an acute pain which may resolve itself after a while. All answers are in the form of dichotomous yes or no responses. The third part of the questionnaire is a more detailed body part-specific questionnaire (neck, low back and shoulder) embedded with some occupational risk factors such as change of jobs or duties, sick leave or prevented from work and hospitalized because of the ache, pain or discomfort as well as duration of symptom over pastime. The fourth section contains four different physical workload factors (neck flexion or rotation, awkward postures, repetitive motion and static postures) to assess the perception of the risk factors causes pain and the potential contribution to NP. Respondents were ask to indicate on a likert scale of 0-4 (0 = no pain, 4 = severe/very severe pain) on how severity of the risk factors contributed to the NP.

The assessment of psychosocial factors was done using the Malay version of Karasek’s Job Content Questionnaire (JCQ) which contains 4 main psychosocial factors (decision latitudes, psychological job demand, social support and job insecurity). A likert-scale of 1 to 4 was used (1=strongly disagree, 2= disagree, 3= agree and 4= strongly agree) to measured all items (decision latitudes, psychological job demand, social support and job insecurity) related to psychosocial risk factors. All psychosocial factors stated above were determined by using median scores as cut off point in which those above median was considered high and those who below median was low. Self-administered SNQ and JCQ were conducted using face to face interview in this study.

Modified SNQ and JCQ were pre-tested the reliability of the questionnaire on 50 rubber tappers with Cronbach’s alpha of 0.689 for SNQ and 0.628 for Malay version of JCQ. The reason of Cronbach’s alpha for Malay version were low is because of low education level among the rubber tappers and face to face explanation is needed in order they understand the question been asked. However, the content in modified SNQ and JCQ questionnaire were still maintain the original content.

### 2.3 Statistical Analysis

Statistical Package for Social Science (SPSS) version 16 was used to calculate the means and standard deviations for continuous data with normal distribution, median and IQR for not normally distributed data. For categorical data, frequency and percentage was used. Chi-square test was applied to determine the risk factors contributing to NP. Simple logistic regression was performed with “enter” method to assess the most affected risk factors for NP. Multicolinearity was adopted to check the interaction between the independent variables. Multiple logistic regressions was performed to assess the independents and interaction effect of all the personal characteristics, physical workloads and psychosocial factors and control for potential confounding variables (i.e. age, amount of trees tapped per day, working experience etc) in which *p* values < 0.05 were included in the model.

### 2.4 Study Ethic

This study was approved by Ethic Committee of Faculty of Medicine and Health Sciences, Universiti Putra Malaysia on 2^nd^ July 2008 and FELDA Headquarter on 31^st^ March 2008. The data was collected with written consent from respondents.

## 3. Results

As shown in Table 1, mean age of respondents was 53.01 ± 4.90 years and the age ranges from 39 to 66 years old. Approximate 67.3% (282) of them aged between 49 to 58 years old. Majority of the respondents (95.9%) were Malay and married (99.35%). In terms of educational level, 47.3% of the respondents achieved education up to Standard 6 level (Primary school) with only 9.3% obtained SPM (Certificate of Secondary Education) / STPM (Certificate of Higher Education) level. The mean BMI was 24.96 ± 3.85 kg/m² and 51.1% of the respondents were within ideal BMI whereas 37.9% were overweight and 7.9% were obese. 54.2% of the respondents were smokers. The mean length of working was 23.56 ± 7.16 yrs and 51.1% of them work between 21 to 30 yrs whereas 3.1% of them work less than 10 years. There are 342 respondents work less than 5 hrs per day. The median of trees tapped per day was 200 ± 150 trees and 27.4% of them were able to tap between 200 to 299 trees per day. For psychosocial factors, majority of respondents in this study had low level of social support (71.4%), psychological demand (65.6%), decision latitude (56.3%) and job insecurity (52.5%) (Table 2).

**Table 1:** Personal details of respondents (n = 419)

Socio-demography	n	%	Mean ± s.d.	Median ± IQR
**Age Group (yrs)**			53.01 ± 4.90	
**39 – 48**	78	18.6		
**49 – 58**	282	67.3		
**≥ 59**	59	14.1		
**Ethnic**				
**Malay**	402	95.9		
**Non-Malay**	17	4.1		
**Marital status**				
**Married**	416	99.3		
**Single/Widow**	3	0.7		
**Education level**				
**< Standard 6**	92	22.0		
**Standard 6**	198	47.3		
**PMR/SRP**	90	21.5		
**SPM/STPM**	39	9.3		
**BMI (kg/m²)**			24.96 ± 3.85	
**< 18.50**	13	3.1		
**18.50 - 24.99**	214	51.1		
**25.00 - 29.99**	159	37.9		
**> 30.00**	33	7.9		
**Smoking habit**				
**Yes**	227	54.2		
**No**	192	45.8		
**Length of working (yrs)**			23.56 ± 7.16	
< 10	13	3.1		
11 to 20	143	34.1		
21 to 30	214	51.1		
31 to 40	35	8.4		
41 to 50	14	3.3		
**Working hours a day (h)**			4.49 ± 1.47	
< 5	342	81.6		
> 5	77	18.4		
**Trees tapped per day**			200 ± 150	
< 200	217	51.8		
200 – 399	114	27.2		
400 – 599	67	16.0		
≥ 500	21	5.0		

**Table 2:** Frequency of psychosocial factors

Psychosocial Factors	n (%)	Median (IQR)
**Social Support**		24 (3)
Low	299 (71.4)	
High	120 (28.6)	
**Psychological Job Demands**		30 (5)
Low	275 (65.5)	
High	144 (34.4)	
**Job Dissatisfaction**		0.2 (0.23)
Low	269 (64.2)	
High	150 (35.8)	
**Decision Latitude**		62 (8)
Low	236 (56.3)	
High	183 (43.7)	
**Depression**		0.15(0.19)
Low	225 (53.7)	
High	194 (46.3)	
**Job Insecurity**		6 (3)
Low	220 (52.5)	
High	199 (47.5)	

**Figure 1:**
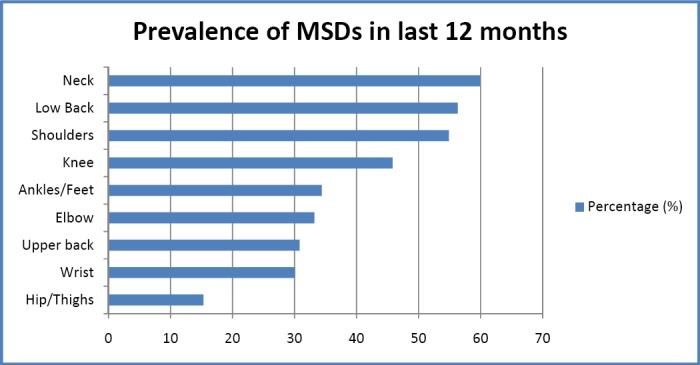
Prevalence of MSDs in last 12 months

Figure 1 showed the prevalence of MSDs in different body region during last 12 months. The most common affected body region among respondents was NP (59.9%) followed by low back pain (56.3%), shoulders pain (54.9%), knee pain (45.8%), ankles/feet pain (34.4%), elbow pain (33.2%), upper back pain (30.8%), wrists pain (30.1%) and hip/thighs pain (15.3%). However, out of 419 respondents, 37.7% of them sort medical advice due to low back pain followed by neck pain and shoulder pain, 33.7% and 33.4% respectively.

Bivariate analysis showed that neck region had significant association with age (χ²= 14.732, *p*= 0.001), working hrs per day (χ²= 4.107, *p*= 0.043), neck flexion or rotation (χ² = 150.043, *p* = 0.001), awkward postures (χ² =81.147, *p* = 0.001), repetitive motion (χ² =52.569, *p* = 0.001), static postures (χ² =40.954, *p* = 0.001) and job insecurity (χ²= 5.539, *p*= 0.019). Other personal characteristic factors (ethnic, marital status, education level, BMI, smoking habit, amount trees tapped per day and working yrs) and psychosocial factors (decision latitudes, psychological demands and social support) showed no significant association with NP.

Simple logistic regression found NP had significant associations with only 2 selected risk factors: age (39-48 yrs) (OR = 3.19, 95% CI 1.29 – 7.90) and neck flexion or rotation (OR = 9.08, 95% CI 5.09 – 16.17). Binary logistic regression with both stepwise forward and backward elimination showed the risk factors which were significant to the model were age (OR= 3.92, 95% CI 1.61 – 9.58), neck flexion or rotation (OR= 9.52, 95% CI 5.55 – 16.32), awkward postures (OR=2.23, 95% CI 1.29 – 3.86) and static postures (OR= 1.86, 95% CI 1.10 – 3.14). The standard error and the correlation were relatively small for these 4 independents risk factors with no interaction effect among them in the model. Other selected factors such as working hours per day, repetitive motion and job insecurity were not significant risk factors for NP (Table 3). Hosmer and Lemeshow Goodness-of-Fit test showed not significant (*p*=0.944), which indicated that the model fitted well and 44.3% of the variation in the outcome variable is explained by this logistic model (Nagelkerke R² = 0.443).

**Table 3:** Neck Pain and Associated Factors by Simple Logistic Regression and Multiple Logistic Regression

Variables	Simple Logistic Regression			Multiple Logistic Regression		
	b	Crude OR (95%CI)	p	b	Adjusted OR (95%CI)	p
**Age (Years)** **39 – 48** **49 – 58** ≥59**	1.160 0.522	3.19 (1.29 - 7.90) 1.69 (0.81 – 3.51)	0.012* 0.162	1.367 0.589	3.92 (1.61 – 9.58) 1.80 (0.88 – 3.70)	0.003* 0.108
**Static Postures** **Yes** No pain**	0.413	1.51 (0.86 – 2.67)	0.155	0.619	1.86 (1.1 – 3.14)	0.021*
**Awkward Postures** **Yes** No pain**	0.590	1.80 (0.99 – 3.28)	0.053	0.802	2.23 (1.29 – 3.86)	0.004*
**Neck flexion or rotation** **Yes** No pain**	2.206	9.08 (5.09 – 16.17)	0.001*	2.253	9.52 (5.55 – 16.32)	0.001*

** Reference category

* p is significant when p<0.05

Backward LR Multiple Logistic Regression model was applied Multicollinearity and interaction term were checked and not found Homers-Lemeshow test, (p= 0.944) and Nagelkerke R Square 0.443 were applied to check the model fitness

**Model and equation for Prevalence of Neck Pain and associated factors:**

Log Y = 0.401 + 1.367* Age (39 - 48) + 2.253 * Neck flexion or rotation + 0.802* Awkward Postures + 0.619 * Static Postures

## 4. Discussion

### 4.1 Respondent Background Data

The study population was all male and aged between 39 to 66 years old with more than 20 years working experience. This is in line with a study conducted by Abu Hassan and Abdullah (2003) among male rubber tappers in FELDA Pahang with age range of 23 to 64 years old. The distribution of age and length of working were mainly influenced by FELDA management in year of 1990 where they decide to stop the intake of settlers for all land schemes throughout all the country. Female workers were not included in this study because they form a small portion (20%) in FELDA helping their husband in conducting the tapping work. In addition, work description was different among female and male in FELDA as female mostly keen to be a housewife rather than helping in tapping. Majority of the respondents have less than 5 working hrs per day because some of the rubber trees were old with less yield of the latex and some of them were die off.

51.8% of the respondents tapped the tress in the range of less than 200 trees per day (total of trees for each rubber workers approximately 1000 trees in 10 acres) with the median±IQR of 200±150 trees. However, the number of trees tapped per day was low as compared with a study conducted by Asyraf *et al*. (2007) in which the mean of rubber trees tapped was 395 trees per day. Based on our observation, the reasons for this is due to most of the rubber trees are old and have many roughly plan as compared with young trees; the older the trees, time requirement was more as they need to conduct the rubber tapping process above their head level. On the other hand, physical condition of the respondents will also affect the number of trees that can be tapped per day; respondents that have a good healthy condition are more likely to tap more trees as compared with the weak ones.

### 4.2 Prevalence of NP and MSDs

The results of this study found overall prevalence of MSDs was high (85.7%) among rubber workers. This finding is in line with a study conducted by Holmberg *et al*. (2002) found 918 (90.6%) reported MSDs and indicating that the odds of reporting musculoskeletal problems were 51% higher among farmers than non-farmers. The highest prevalence of MSDs found in this study was NP with prevalence of 59.9%, followed by LBP (56.3%) and SP (54.9%). These findings was consistent with a study conducted by Kuorinka *et al*. (1987) in which NP, LBP and SP were the most common and predominantly occurring prevalence of MSDs. In the year of 2007, a study conducted by Asyraf *et al*. reported the prevalence of NP was 48.8%. In contrast, Rosecrance *et al*. (2006) reported the prevalence was low among Kansas farmers and Ismail (2007) reported the prevalence as 36.3% among palm oil plantation workers in Sabah. The contrary findings in this study were difficult to conclude as there has been very limited documentation and literature on MSDs and neck MSDs in the agriculture industry (Kermit and Susan, 2007).

Based on observation in rubber field on the tapping process, high prevalence of NP among the rubber workers could be due to the rubber trees condition. Most of the rubber trees are old and need to be tapped above their head level and hence extreme force was required to extend their postures mainly on neck, shoulders and forearm area. This will induce a high risk to the neck pain.

### 4.3 Association between NP and Personal Characteristics

This study found a significant inverse association between age and NP. Binary logistic regression showed those with age between 39-48 years was 4 times more likely to have neck pain as compared with age group of 48 – 58 and more than 59 years old rubber workers. The finding was in contrast with case control study by Hartman *et al*. (2006) on risk factors for sick leave due to MSDs among self-employed Dutch farmers showed that risk factors for musculoskeletal pain (neck, back, shoulder or upper extremity pain) was increased with age and also with sick leaves due to this disorder.

In this study, young workers was at greater risk to develop neck pain as compared with older age workers because older workers employed foreign workers in helping them to tap the rubber trees in FELDA. Based on observation, most of the rubber tapping process was only conducted in less than 5 hours per day; normally from 7am to 11am. Young rubber workers also involved in helping their parents in cow and sheep farming in the afternoon. The explanation was not uncommon as other study conducted by Park *et al*. (2001) showed older farmers in Iowa performs less physical labor leaving the difficult high force tasks to younger worker. In addition, older rubber workers may have known and learned the right technique of rubber tapping process to avoid pain and discomfort as compared with the young workers who do not have experience in tapping process. “Survivor bias” occurs which means only healthy workers able to continue the current work after the others have dropped out.

There were no significant association found between NP and amount of trees tapped per day. It may due to other physical workload factors such as geographical terrain and neck flexion or different awkward postures were the dominant risk factors for NP. NP had no significant association with working hrs per day, BMI, ethnic, marital status, education level, smoking habit, length of working and trees tapped per day. There was limited literature on association between NP and personal characteristics factors. Nevertheless, a study conducted by Abu Hassan and Hasbullah (2003) on low back pain among rubber tappers show no significant association with socio-demographic factors (educational level, length of working, working experience and pain on low back in previous years).

### 4.4 Association between NP and Physical Workloads

This study found that all the physical workload factors (repetitive motion, static posture, awkward posture and neck flexion or rotation) have significant association with NP. The findings of our study were in lieu with several studies that conclude repetitive and monotonous work tasks, awkward posture and neck extension were main risk factors for NP (Meyers *et al.*, 2000; MOA and MPIC, 2005; Ohlsson *et al.*, 1995). Multivariate analysis found neck flexion or rotation, awkward and static postures were the main risk factors for NP where repetitive motion showed no significant association.

Binary logistic regression models showed that respondent who fall in the age of 39 to 48 years old had greater risk of NP when conducted rubber tapping tasks with neck flexion, awkward and static postures.

**Figure 2:**
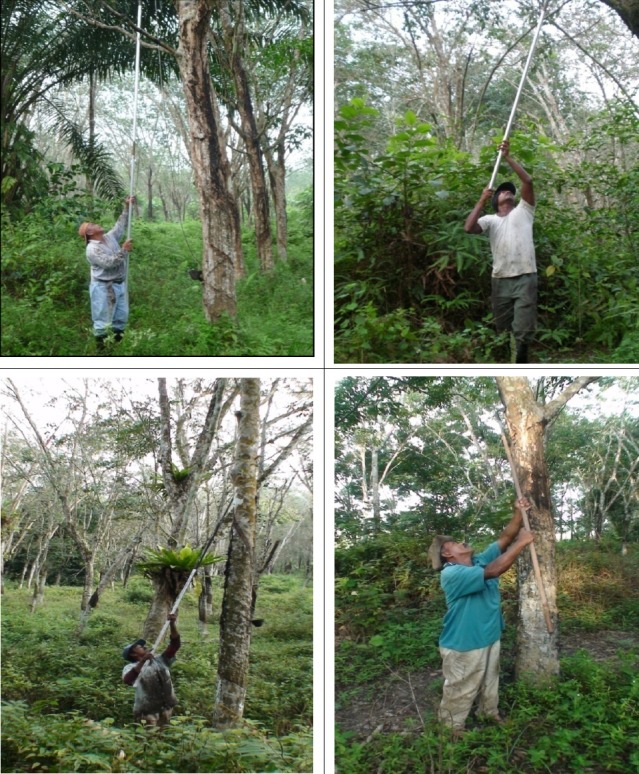
Neck flexion or rotation, awkward and static postures while conducting tapping process

Rubber tapping processes for trees up to 6 meters require the respondents to perform it above their head level. Hence, extreme force required and they need to extend their postures mainly on neck for long duration during tapping task and poses higher risk to develop NP (Figure 2). Similar explanation was elaborated by Meyers *et al*. (2000) which stated neck extension occurs when the farm workers do tasks above their heads in tasks such as fruit harvesting and wine grape pruning.

Awkward postures for example reaching above, twisting, forward or backward bending were significant risk factors for NP. Respondents were forced to flex and rotate their head in order to tap the rubber trees that were above their head level, it causes significant deviation from their neutral position. During tapping process it lead to further over extension of neck muscle. Muscle in this position would not work effectively, required more energy and fatigue faster (Figure 2).

Static postures found significant factors for NP as the rubber workers force to work and tapped the trees above head level for long duration. Neck region required to stay in static postures for average 1 to 2 minutes based on observation to ensure the latex was flow in the right position into the cup. These might increases the load and forces lead to muscle fatigue for neck pain.

### 4.5 Association between NP and Psychosocial Factors

Job insecurity was associated with a decrease in both physical and mental health status among wide range of occupation, organizations and positions among 729 Norwegian employees (Storseth, 2006). Study among female flight attendants by Hyeonkyeong *et al*. (2008) found that higher feelings of job insecurity (OR = 1.35, 95% CI = 1.09 to 1.68) were associated with a higher incidence of lower-back. Contrary to our finding, we found that job insecurity was associated with NP but not significant with LBP. Different population study may report different sensation of pain in their body parts. A study conducted by Lundberg *et al*. (1994), Melin and Lundberg (1997) found that job insecurity may lead to “physiological vulnerability” of muscles and to the sensation of pain. Nevertheless, further multivariate analysis in this study showed there was no significant association of job insecurity and NP. This shows that other risk factors such as physical workload were dominant associated risk factors for NP rather than psychosocial factors. The specific route or routes by which psychological stress/strain might influence MSS is a matter of debate (Zara, 2008).

## 5. Conclusion

The high prevalence of MSS shows that MSS is a significant problem among rubber workers. NP showed the highest prevalence as compared with other body regions. This study found that age had inverse relationship with NP. Among ergonomic factors neck flexion, awkward postures, repetitive motion and static postures were found significant relationship with NP. Binary logistic regression revealed age, neck flexion or rotation, awkward and static posture was the most affected risk factors for NP. This study recommended that detail study should be carry out on each ergonomic factors with objective measurement tools which include anthropometry measurement and biomechanic in order to comprehensively understand the mechanism of MSD. In addition, management should instituted health promotion activities and guidelines to empower workers to minimize the risk and better quality of life especially for young workers.
